# Vitrimeric shape memory polymer-based fingertips for adaptive grasping

**DOI:** 10.3389/frobt.2023.1206579

**Published:** 2023-07-12

**Authors:** Seyedreza Kashef Tabrizian, Walter Alabiso, Usman Shaukat, Seppe Terryn, Elisabeth Rossegger, Joost Brancart, Julie Legrand, Sandra Schlögl, Bram Vanderborght

**Affiliations:** ^1^ Brubotics, Vrije Universiteit Brussel (VUB) and Imec, Brussels, Belgium; ^2^ Polymer Competence Center Leoben GmbH, Leoben, Austria; ^3^ Physical Chemistry and Polymer Science (FYSC), Brussels, Belgium

**Keywords:** adaptive grasping, shape memory polymer, shape adaptive fingertip, manipulation, additive manufacturing

## Abstract

The variability in the shapes and sizes of objects presents a significant challenge for two-finger robotic grippers when it comes to manipulating them. Based on the chemistry of vitrimers (a new class of polymer materials that have dynamic covalent bonds, which allow them to reversibly change their mechanical properties under specific conditions), we present two designs as 3D-printed shape memory polymer-based shape-adaptive fingertips (SMP-SAF). The fingertips have two main properties needed for an effective grasping. First, the ability to adapt their shape to different objects. Second, exhibiting variable rigidity, to lock and retain this new shape without the need for any continuous external triggering system. Our two design strategies are: 1) A curved part, which is suitable for grasping delicate and fragile objects. In this mode and prior to gripping, the SMP-SAFs are straightened by the force of the parallel gripper and are adapted to the object by shape memory activation. 2) A straight part that takes on the form of the objects by contact force with them. This mode is better suited for gripping hard bodies and provides a more straightforward shape programming process. The SMP-SAFs can be programmed by heating them up above glass transition temperature (54°C) via Joule-effect of the integrated electrically conductive wire or by using a heat gun, followed by reshaping by the external forces (without human intervention), and subsequently fixing the new shape upon cooling. As the shape programming process is time-consuming, this technique suits adaptive sorting lines where the variety of objects is not changed from grasp to grasp, but from batch to batch.

## 1 Introduction

Manipulating objects by two finger robotic grippers is one of the most common tasks in robotics (Birglen and Schlicht, 2018). Many efforts have been made to develop more robust grippers to manipulate objects of various shapes and sizes, using different technologies such as complex multi-DOF robotic hands (Kim et al., 2021), soft robotics (Shintake et al., 2018), shape-adaptive linkage coupling mechanisms (Kashef et al., 2020), and origami designs (Kan et al., 2020). Nowadays, with the ongoing development of smart and stimuli-responsive materials, elegant solutions have been presented for various robotics problems, such as manipulation, locomotion and actuation (Chen et al., 2020).

One of the most promising types of smart materials are the shape memory polymers. They have the ability to return to their original shape after being programmed to a new temporary shape, making them suitable for applications where adaptation and shape-changing capabilities are advantageous, such as in grippers (Linghu et al., 2020; Chen and Peng, 2021). Different types of shape memory polymers exist, with the one-way heat-activated property being the most common type that provides reliable function (Scalet, 2020). Using shape memory polymers in robotics applications can reduce the complexity of the systems and integrate the motion actuation in the structure of the robot (Chen et al., 2020; Scalet, 2020; Xia et al., 2021).

However, one-way shape memory requires two external stimulations to perform their function. First is the heat-cool cycle needed for phase shifting of the material, and the second is the application of an external load needed for deforming the material, both of which are required for every shape programming (Scalet, 2020). In addition, polymers generally have poor thermal conductivity, which hinders their adoption in real industrial applications where a high frequency of motion is needed (Linghu et al., 2020; Schönfeld et al., 2021). The issue lies in cooling the material, which is necessary to fix a newly programmed shape. Even with the integration of a cooling system (Zhang et al., 2019), it still takes several seconds to go through the shaping process. Furthermore, shape memory polymers suffer from low force generation. This restricts their range of applications when used as an active and actuation element (Schönfeld et al., 2021). Considering these limitations, the authors argue that utilizing shape memory polymers to make an entire gripper is not an appropriate selection of application. It limits the range of the objects that can be manipulated and the potential use cases where the gripper can meet the manipulation requirements, e.g., the required frequency of the pick-and-place task. Addressing these issues needs more research on optimizing the thermal (Bartlett et al., 2017), and mechanical properties of the materials (Zhang et al., 2022).

**FIGURE 1:**
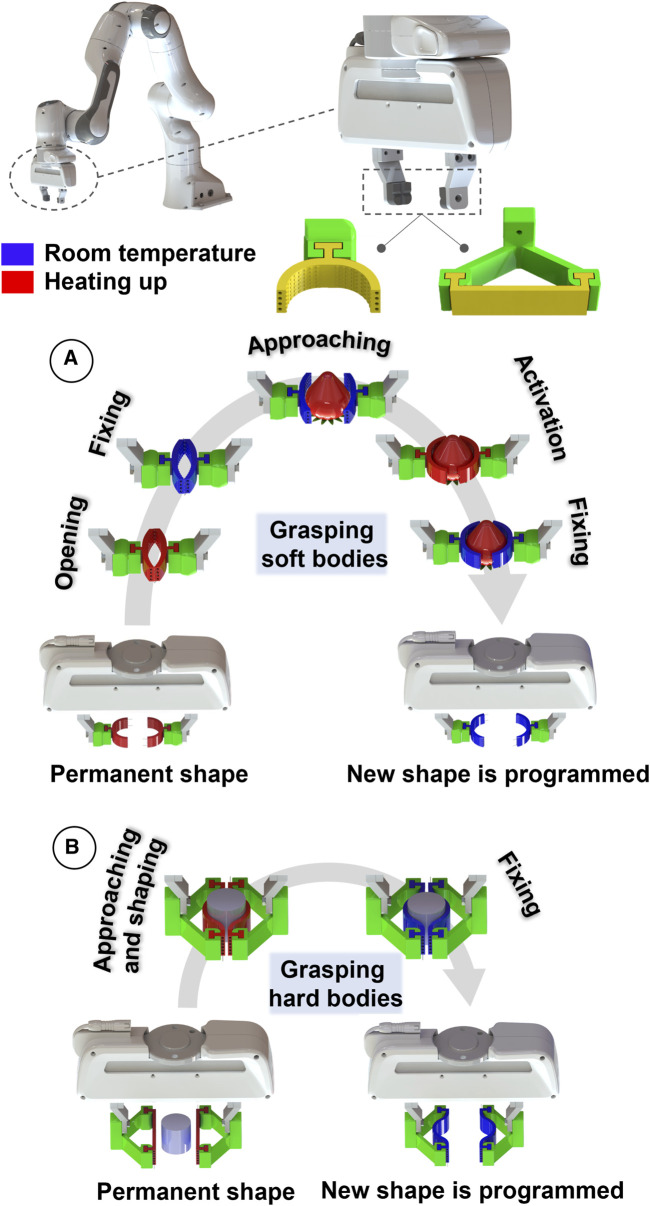
Shape adaptation methodology of the two SMP-SAFs. **(A)** The curved SMP-SAF is used for grasping soft bodies. There is a pre-programming step in which the fingertips are straightened by heating them up, pressing against each other followed by cooling them down. Shape adaptation also includes a heat-cool cycle starting with approaching the fingertips to the objects, hearting for shape memory activation and cooling for new shape fixing. **(B)** The straight one is applied for grasping hard bodies in which the fingertips are first heated up, then pressed against the object to take its shape and cooled down to fix the new shape.

Despite the mentioned challenges associated with developing grippers out of shape memory polymers, the materials still hold the potential for producing shape-adaptive fingertips attached to a robotic gripper, with the issues being addressable at the system or application level. This means that the shape memory materials are used as a shape-adaptation element rather than an actuation element. In the case of utilizing shape memory polymers as fingertips, the shape transition (to provide a shape-adaptive contact with the object) is only needed when an object with a new shape is going to be manipulated, not for each pick-and-place task. In many cases of sorting and assembly lines, the shape of the objects is not changed frequently but from batch to batch. In addition, the robotic gripper can be appropriately selected to meet the required speed and load for the manipulation task. Furthermore, the gripper itself can serve as the external load required for the shape programming process (Figure 1). Researchers have also published other design ideas for a shape-adaptive fingertip, for example, by exploiting the principle of granular jamming (Hou et al., 2019; Lee et al., 2021), magnetorheological elastomers that can shift from a soft to a solid-like state by applying a magnetic field (Choi et al., 2020), and active soft cavities embedded in soft matter that can change the shape of the soft matter fingertip for a firmer grasp (He et al., 2020). Compared to shape memory polymer property, which is seen in many polymers, these alternatives are more complex in design and fabrication. In the case of the jamming principle and using soft cavities, they are vulnerable to damage by sharp objects.

In recent years and more specifically in the domain of soft robotics, different types of grippers have been presented that offer shape-adaptive gripping ability (Mosadegh et al., 2014; Manti et al., 2015). Research on hand-like soft grippers to enhance grasp efficiency, shape-conformability and load-carrying capacity is also a trending topic. To achieve this, a common strategy is to tune the stiffness, ranging from soft for shape conformability to rigid for load-carrying enhancement. This shift in stiffness can be attained by exploiting the principle of jamming (Wei et al., 2016; Bakarich et al., 2022), or multi-phase materials (Zhang et al., 2019; Wang et al., 2020; Zhuo et al., 2020). Compared to hand-like grippers, universal/enveloping grippers show a higher level of shape conformability and there exist multiple enveloping mechanisms, e.g., universal jamming gripper (Brown et al., 2010), universal origami-based gripper (Li et al., 2019), universal accordion structure-based gripper (Hao et al., 2021), and scooping-binding parallel gripper (Wang et al., 2021). Linghu et al., have made a simple structure universal gripper based on shape memory polymer material that is capable of grasping arbitrarily shaped objects by heating the material up, and subsequently pressing it on top of the objects while cooling it down to lock them (Linghu et al., 2020). This gripper is suitable for gripping hard objects but has the drawback of slow response time (in the order of minutes) resulting from the heating-cooling cycle that is needed for each grasp.

Despite that, parallel two-finger industrial grippers are still the most common type of grippers used in industry (Birglen and Schlicht, 2018). They have a simple structure, robust in their function and offer facile control. However, it is always desired to provide shape-adaptive contact with objects to grasp hard-bodied objects, but more importantly to manipulate delicate and fragile ones. Thus, a secure grasp can be guaranteed by a reduced local gripping force, which may contribute to preventing damage to the products. Moreover, better enveloping the objects creates a more stable and secure grasp during high-speed manipulations. In view of this, the need arises to fabricate shape-adaptive fingertips for parallel grippers with the ability to take the shape of at least the most common geometries of objects in pick-and-place tasks, like spherical, cylindrical, pyramidal and cubic.

Herein, we present two designs of shape-adaptive fingertips, for adaptation to delicate objects and hard bodies (Figure 1). They are attached to a parallel gripper, can change their shape based on the geometry of the target objects and retain the new shape until the definition of a new task: manipulating other batches of certain objects with a different outer surface profile. They are made out of a photocurable vitrimeric network manufactured by means of Digital Light Processing. In this demonstration, the system is independent of human intervention for the shape programming cycle. The SMP-SAFs can be heated via Joule-effect of the embedded electrically conductive wires (manual heating by a heat gun is also possible), and reshaped upon contact with the objects or by force of the parallel gripper (Figure 1).

This is in line with fifth industrial revolution to make the production and manufacturing processes adaptable to changes, combine customized systems with mass production and keep speed and flexibility in assembly and sorting lines.

## 2 Materials and methods

### 2.1 Shape adaptation methodology

Based on the properties of the objects, two different models were made for the SMP-SAF (Figure 1). The first one, intended for handling delicate and fragile objects, consists of two SMP-SAFs printed with a curved permanent shape. First, they are heated above T_g_, then pressed together using the force of the jaw gripper, and straightened. After cooling below T_g_, the straightened state is retained. Next, the gripper approaches the object and makes the first contact with it. Reheating the fingertips activates the shape memory effect, causing them to return to the permanent shape, the wings are closed until making contact with the object and take the profile of that. Having more contact points is beneficial when handling delicate items. Fewer contact points can result in larger contact forces that can bruise the items. This new shape can be fixed by cooling the SMP-SAFs down to room temperature. Other objects with similar shapes can then be manipulated more efficiently. The SMP-SAFs will go back to the fully bent position by reheating, setting up for a new shape programming.

The second approach consists in grasping of hard bodies where the fingertips are printed in straight mode, already suitable for handling objects with a flat surface profile. At room temperature, the fingertips are quite stiff and remember the printed shape as the permanent shape. To adapt to other geometries, the SMF-SMFs are first heated up to the glass transition temperature (T_g_) of 54 °C, becoming flexible, and then approach the object. The SMP-SAFs adapt to the outer form of the object through contact forces, and then cool down to room temperature to retain the new shape. Thereafter, the SMP-SAFs are ready to manipulate other objects having a similar outer surface profile as the grasped one. Re-heating the fingertips will bring them back to the permanent mode.

The curved fingertips can also be used to adapt to hard objects and manipulate them. However, the process of adapting the curved fingertips involves two shape programming steps - first opening and then adaptation - which can be more complex than the procedure required for straight fingertips (as shown in Figure 1). On the other hand, the design of straight fingertips carries a higher risk of damage to delicate objects since the fingertips must be pressed against the object for shape adaptation. These considerations have led us to introduce two different designs, each of which can be selected based on the application and the type of objects that are being manipulated.

### 2.2 Photo-curable vitrimeric resin

Vitrimers are a special subclass of polymers, characterized by a dynamic covalent network (Winne et al., 2019). While they possess the properties of conventional thermosets, they can be reprocessed and reshaped above a characteristic transition temperature, where the dynamic bond exchange proceeds at a sufficiently fast rate to promote a macroscopic reflow (Denissen et al., 2016). This feature endows them with some remarkable properties, such as the ability to be reprocessed, welded and to self-heal damage (Alabiso and Schlögl, 2020). A currently available review covers the topic in detail (Alabiso and Schlögl, 2020).

**FIGURE 2:**
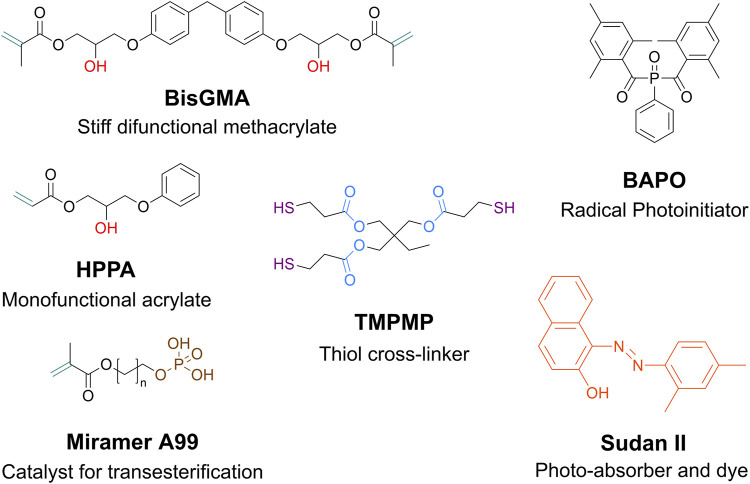
Chemical structure of the components in the photo-curable thiol-acrylate network (Alabiso et al., 2021). The carbon double bonds (-C=C-, dark green) react with the thiols (-SH, purple). The reaction is initiated by BAPO as a visible-light photoinitiator. Hydroxy groups (-OH, red) and esters (-COO-, light blue) participate in the dynamic bond exchange, i.e., transesterification. Miramer A99 acts as a catalyst and promotes the stiffening of the network. Sudan II is a photo-absorber and improves the printing quality by stabilizing the resin bath.

In the case of this study, the application of the presented shape-adaptive fingertips requires a material with two main features. First, the shape memory property is needed for shape adaptation and fixing different temporary shapes to accommodate diverse target objects. Second, the T_g_ needs to be sufficiently higher than room temperature to retain any new programmed shape in normal working conditions. We chose the material for this study based on our recent work, which featured shape memory-assisted self-healing of dynamic thiol-acrylate networks (Alabiso et al., 2021). The authors introduced a series of resins with an appealing combination of properties, such as toughness, tunable T_g_ as a function of the cross-linker content, shape memory and self-healing via transesterification (i.e., exchange reaction between -OH groups and esters) (Alabiso et al., 2021). Here, we chose the resin with 14 mol% content of thiol cross-linker, as we observed it was less prone to brittle failure during shape programming. Trimethylolpropane tris(3-mercaptopropionate) (TMPMP) was bought from Worlée Chemie GmbH (Germany). The organic methacrylate phosphonate Miramer A99 was purchased from Miwon Specialty Chemical (Korea). All other chemicals were purchased from Sigma-Aldrich (United States of America) and used as received (Figure 2). From a chemical point of view, the formulation is based on a photoinitiated reaction between the thiol moieties (-SH) of a trifunctional cross-linker (TMPMP) and the carbon double bonds (-C=C-) of the (meth)acrylate units present in BisGMA and HPPA (Figure 2). In the original study, the behaviour of Miramer A99 as a catalyst for transesterification was investigated. It was moreover observed that the presence of an organic phosphonate in the network may cause a stiffening of the material upon thermal treatment, consequently shifting the glass transition to higher temperatures (Rossegger et al., 2021). Hence, we proceeded with a treatment step (4 h at 180 °C in a regular oven) to attain the desired T_g_, though compromising the healing ability of the material. Therefore, we did not focus herein on the self-healing ability, but rather primarily on the benefits from shape memory property. Lastly, unlike the previous work (Alabiso et al., 2021), we used the Digital Light Processing technique to 3D-print the resin. For this purpose, adding 0.02 wt% Sudan II as a photo-absorber turned out to be beneficial for the printing process. The authors previously reported that this family of thiol-acrylate vitrimers is well-suited for Digital Light Processing of 3D-printed soft devices, which could potentially make the manufacturing of soft robotic parts easy and customizable (Rossegger et al., 2021; Shaukat et al., 2021).

### 2.3 Fabrication

**FIGURE 3:**
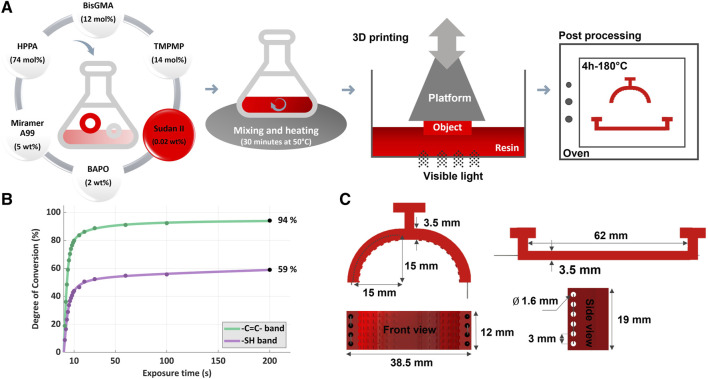
Processing of the SMP-SAFs. **(A)** To create the 3D printed fingertips, the process begins by synthesizing the resin through the addition of chemical components and stirring. This resin is then printed using the Digital Light Processing technique. Once printing is complete, the parts undergo thermal treatment to attain the desired Tg. **(B)** FTIR analysis on the photocurable resin shows the conversion of the thiol groups (-SH) and (meth)acrylate groups (-C=C-) in the network based on the illumination time. This provides information on the exposure time needed to cure the resin. **(C)** Dimensions of both the curved and straight fingertips.

Processing of the presented fingertips comprises a few facile steps (Figure 3A). The photopolymer resin is first made by synthesizing the chemical compounds followed by stirring for a few minutes at a mild temperature (i.e., 30 min at 50 °C). The resin is then poured into the vat of the printer and the printing is started. Finally, the parts are detached from the print bed, washed and postprocessed for 4 h at 180 °C. The red color of the fingertips comes from Sudan II, although this color sometimes disappeared (Sudan oxidized away) after the thermal treatment. The exposure times of the bottom layer and the following layer were set to 65 s and 18 s, respectively. These settings were determined in light of a kinetics study on the resin via Fourier Transform InfraRed (FTIR) analysis (Vertex 70 spectrometer (Bruker, United States) in conjunction with OPUS v7.5 software) (Figure 3B) (Alabiso et al., 2021). It is a technique that uses infrared absorption spectra to analyze the chemical bonds present in a molecule. By generating a molecular fingerprint through the spectra, FTIR enables the screening and scanning of samples to identify various components with distinct profiles. To ensure the adhesion of the printed part to the print platform, four bottom layers were printed. The lift distance, the lift and the retract speed were set to 6 mm, 3 mm/s and 2 mm/s, respectively. Despite the presence of the internal holes within the fingertips, it is still feasible to fabricate them through molding and casting. However, Using additive manufacturing for producing the fingertips not only provides greater design freedom, but also addresses the challenge of low penetration depth of light during the curing process of photo-curable resins. By employing additive manufacturing techniques, the fingertips can be fabricated in a layered manner, allowing for more efficient and thorough curing of the resin. Nevertheless, it is important to acknowledge that upscaling remains a challenge in the field of additive manufacturing.

The performance of the shape memory polymer-based actuators/parts directly depends on the geometry. Increasing the dimension of the fingertips leads to an increase in heating and cooling time or related energy consumption. In addition, less adaptability is observed with thicker fingertips (at least in the case of the straight fingertip design), and the shape programming process requires applying a greater external load. Conversely, a larger size provides greater shape memory force and more strength against structural failure. Here, the focus is on utilizing the shape memory property for adaptability, not as an actuating element. As a result, being thinner was given priority over being thicker. There were some practical design limitations as well. One of them is the integrated holes in the structure of the fingertips. The thickness of the fingertips should be large enough to accommodate holes of 1 mm–2 mm to easily pass the wires after printing. All in all, the thickness of the SMP-SAFs is 3.5 mm and the holes with 1.6 mm in diameter and center distance of 3 mm were designed to accommodate the electrically conductive wires for heating via Joule effect. Dimensions of both SMP-SAFs are in Figure 3C. With these geomaterial parameters, the maximum load of the Franka Emika two-finger gripper can be used without structural failure of the fingertips.

### 2.4 Temperature-dependent properties

**FIGURE 4:**
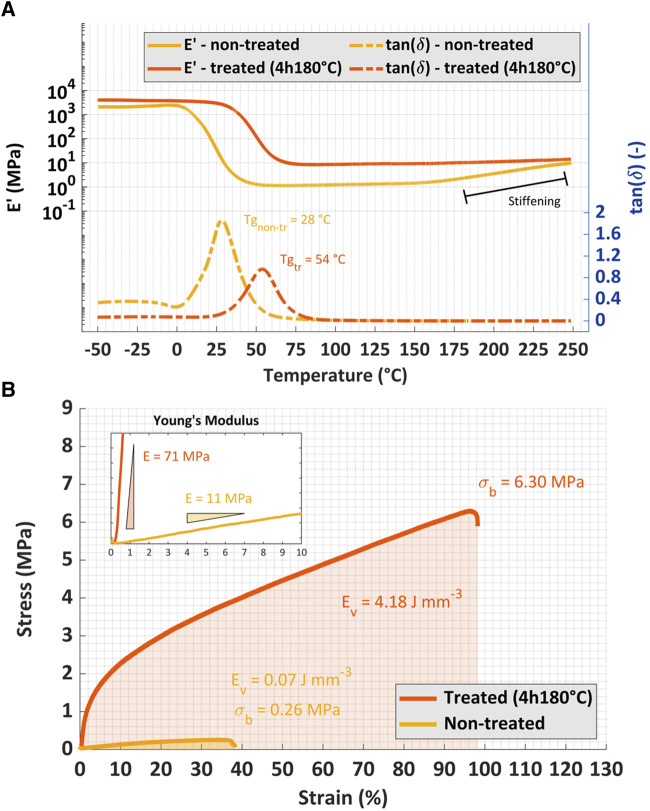
Characterization of the material. **(A)** Dynamic mechanical analysis of the specimen before and after thermal treatment shows a considerable permanent change in the storage modulus as well as the Tg of the material. **(B)** Tensile test on the specimens before and after treatment illustrate an almost 25 times increase in the Young’s modulus upon treatment.

To measure the T_g_ of the material after 3D-printing, rectangular specimens with a length, a width and a thickness of 15 mm, 4 mm and 1.5 mm, were printed and subjected to Dynamic Mechanical Analysis (TA Instruments Q800 DMA) (Figure 4A). Measurements were carried out on both fresh and thermally treated specimens to evidence the shift in T_g_ after treatment. During the DMA tests, the applied amplitude and frequency were set at 0.01% and 1 Hz, respectively. Similarly, tensile testing was performed to characterize the Young’s modulus of the 3D-printed parts (Figure 4B). Rectangular specimens, 30 mm long, 10 mm wide and 2 mm thick, were subjected to elongation at a rate of 1%/s using a Tinius Olsen tension machine.

### 2.5 Robotics manipulation

A Franka Emika robot manipulator with 7 DoF and a 2-finger parallel gripper as end effector was used to grasp and manipulate objects (Figure 1). The robot can be programmed via the software of Franka Emika Robot System. The fingertips of the gripper are exchangeable, to our developed SMP-SAFs, which replace the typical pads. The embedded wires in the SMP-SAFs for joule-heating are nickel-titanium wires that can heat the SMP-SAFs above T_g_ with about 6 V.

## 3 Results

### 3.1 Material properties

Figure 3B presents the results of the FTIR analysis of the printable resin, showing the conversion rate of the thiol (2,569 cm^-1^) groups in purple curve (-SH band) and the (meth)acrylate carbon-carbon (-C=C-) group (1,636 cm^-1^) in green curve (Alabiso et al., 2021). This illustrates the amount of exposure time needed to have the resin cured. Using the conversion plateau starting from 15–20 s as an initial indication of a complete cure, we determined a general exposure time of 18 s for Digital Light Processing by trial-and-error.

To achieve the desired T_g_, thermal post-treatment is needed. Figure 4A illustrates how this treatment shifts the T_g_ of the printed parts from 28 °C to 54 °C. It also shows the change in the storage modulus of the material as a function of the temperature. Many polymers show a viscoelastic property where the storage modulus is the indication of the elastic response of the material and loss modulus is the indication of the viscous behavior of the material (Zhang et al., 2022). As such, the storage modulus is a reasonable approximation of the stiffness of the material. As seen, the thermal treatment permanently changes the storage modulus of the material and makes it stiffer.

The change in mechanical property by the thermal treatment is evident in Figure 4B as well, which presents the results of a quasistatic tensile test: the Young’s modulus of the treated material is 25 times higher than the non-treated one. Furthermore, the toughness drastically increases by nearly 60 times, from 0.07 J/mm^3^ to 4.18 J/mm^3^.

### 3.2 Shape memory efficiency

**FIGURE 5:**
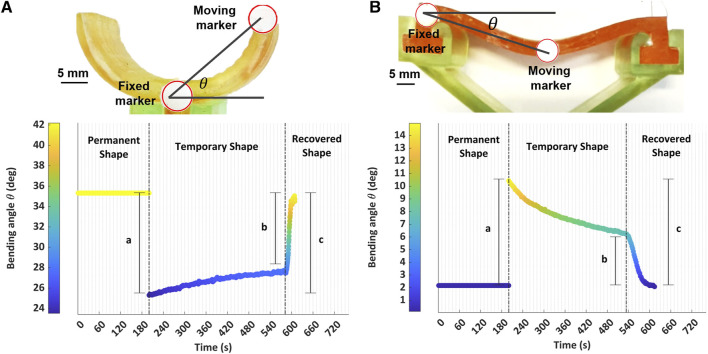
Shape memory efficiency of the SMP-SAFs. **(A)** Initial bending angle equals 42°. The wings are then programmed until a bending angle of 24° and are kept for almost 6 min followed by reprogramming to the permanent shape. **(B)** Initial bending angle is 0°. The fingertip is first programmed until 14° and kept for almost 6 min. It is then heated up again to recover the initial state. In both subfigures, b/a represents the shape fixing efficiency, whereas c/a corresponds to shape recovery efficiency.

In order to quantify the shape stability of temporary configurations of the SMP-SAFs and the recovery of their permanent shape, we define a shape-fixing efficiency and a shape-recovery efficiency, respectively. For a quantitative study, two white markers are attached to the fingertips while they are programmed from the permanent mode to a temporary shape and recovered to the permanent shape (Figure 5). The position of the markers is tracked during shape programming via video recording and post-processing, using *imfindcircles* function in Matlab (The MathWorks, Massachusetts, US). While one of the markers is in a fixed position, the relative displacement of the other is calculated (Figure 5). The SMP-SAF is reshaped and the position of the markers is compared during the next six minutes with the position immediately after reshaping. This allows the estimation of the shape-fixing efficiency. After reshaping, the SMP-SAF is reheated to recover the permanent mode. Comparing the position of the markers before the first shape programming and after the last shape programming provides information about shape-recovery efficiency. As seen in Figure 5A, the shape fixing efficiency (b/a) and the shape recovery efficiency (c/a) for the curved SMP-SAF are 89.5% and 97.3%, respectively. Those of the straight SMP-SAF (Figure 5B) are 50% and 100%. The lower shape-fixing efficiency of the straight SMP-SAF is related to the geometry. As the two sides of the fingertip are fixed, more stress is needed to deform it. That being the case, there is a strong tendency to recover the permanent shape, and it is more challenging to freeze the relative movement of the chains in the network, which is needed for shape fixing. Apart from geometrical optimization, formulation of a material with higher T_g_ improves the shape fixing efficiency, as the chain movements are better frozen at room temperature. However, this comes along with the drawback of an increased energy consumption for shape programming. In addition, achieving a more homogenous network with a sharper T_g_ transition will reduce the chance of undesired shape recovery and make the shape programming easier to control. Note that the shape-fixing efficiency is far more important for the curved SMP-SAF. If it unintentionally recovers its permanent shape, meaning that the opening of the fingertips becomes narrower, it may fail in grasping objects larger than the opening size.

### 3.3 Grasping delicate objects

**FIGURE 6:**
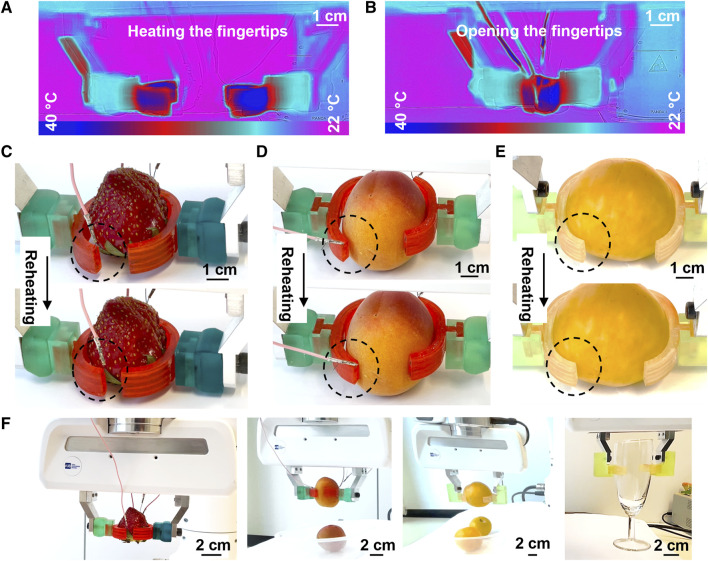
Shape adaptation and grasping with curved SMP-SAFs. **(A)** Heating the fingertips **(B)** and pressing against each other to open the wings. **(C)** shape adaptation to a strawberry by approaching the fingertips to it and activating the shape memory effect. Same approach is performed for **(D)** an apricot and **(E)** a plum. **(F)** Manipulating several of the three fruits and the wineglasses after shape adaptation of the fingertips.

The curved SMP-SAF is designed to handle delicate objects, because its adaptation does not rely on pressing the object against the fingertip (Figure 1). The two curved SMP-SAFs are first heated via joule-heating of the embedded electrical wires (Figure 6A) and are opened and straightened by pressing the SMP-SAFs together using the force of the jaw gripper (Figure 6B). This new shape is fixed by cooling down to room temperature. Next, the gripper approaches a delicate soft object, e.g., a fruit, and the SMP-SAFs touch it. It is important for the robot to carefully approach and touch the objects in order not to bruise them. In this study, it is done by human assistance through visual inspection. However, a high-resolution force sensor or a vision-based grasp planning with the object position detection ability can later be integrated in the system. As seen in Figures 6C–E, the shape memory is then activated by reheating the fingertips, resulting in their envelopment around the fruit, providing a maximal grasping contact area. As a result, the manipulation of objects will be carried out in a more secure way. Figure 6F shows the grasps and manipulation of three different fruits and a wine glass (Supplementary video). Although less adaptable in comparison with the straight SMP-SAF, the curved SMP-SAF has also the ability to be used for grasping hard bodies, e.g., manipulating the glass. This fingertip design is not able to adapt to the shape of objects with no side/diameter larger than 26.5 mm (Figure 3A). Although adaptation to smaller objects is not possible, this does not mean that those objects cannot be gripped by the curved fingertips. They can be straightened and act as typical fingertips to grasp objects with the mentioned size limitation.

### 3.4 Grasping hard bodies

**FIGURE 7:**
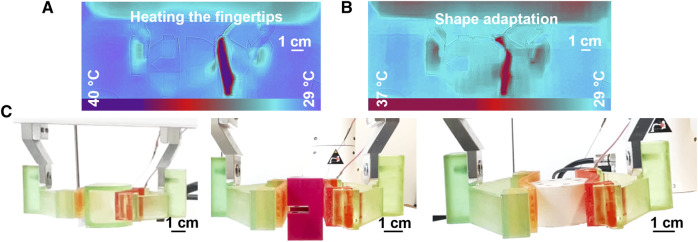
Shape adaptation and grasping with straight SMP-SAF. **(A)** As the object is a half-cylinder, just one fingertip is heated to take the form of the curved side. **(B)** Pressing the fingertips against the object to take its shape. **(C)** shape adaptation and manipulation of the three different geometries including a half-cylinder, a cube and a cylinder.

Three different geometries, namely, a beam, a cylinder and a half-cylinder, were chosen to demonstrate grasping with the straight SMP-SAF. The beam illustrates that the gripper equipped with the SMP-SAF, is able to grasp objects with flat walls, while the cylinder proves that SMP-SAFs can be adapted to grasp curved objects. In addition, the SMP-SAFs can be adapted to more complex shapes as demonstrated by grasping a half-cylinder. As mentioned before, the fingertips can either be activated by Joule effect or by a heat gun. When one or two sides of the object are flat, there is no need for shape programming. In other cases, the fingertips should first be heated (Figure 7A) and adapted to the outer surface profile of the objects by pressing them against each other (Figure 7B). By using this approach, the fingertips can take different shapes of the objects, without the need for replacing them with a new fingertip, every time a series of a new object is manipulated. Figure 7C shows the grasping and manipulation of three shapes, including a cube, a cylinder and a half-cylinder, using the straight SMP-SAFs (Supplementary video). Considering the maximum load of the Franka Emika two-finger robotic gripper to press the fingertips against different objects, this fingertip design can change its form from infinite curvature radius (the straight mode) to 70 mm curvature radius (a deformed shape).

**FIGURE 8:**
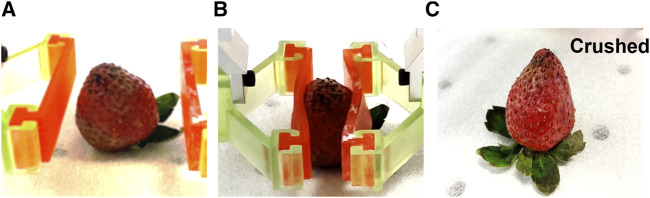
Straight SMP-SAFs damage delicate objects. **(A)** the shape of the strawberry before grasping. **(B)** The fingertips are heated up and press against the object to take its shape. **(C)** The strawberry is crushed as a result of pressing by the gripper.

This grasping strategy can pose a risk to delicate objects. The straight SMP-SAFs adapt to the object through contact forces, which may be too strong for objects that are fragile or delicate. The forces applied by the SMP-SAFs can cause damage or deformity to the object. For instance, Figure 8 shows how a strawberry is crushed when the fingertips are pressed against it to take its shape. Using this approach, it is crucial to consider the fragility and delicacy of the object compared to the softness of the fingertips in the heated state.

### 3.5 Grasp stability analysis

**FIGURE 9:**
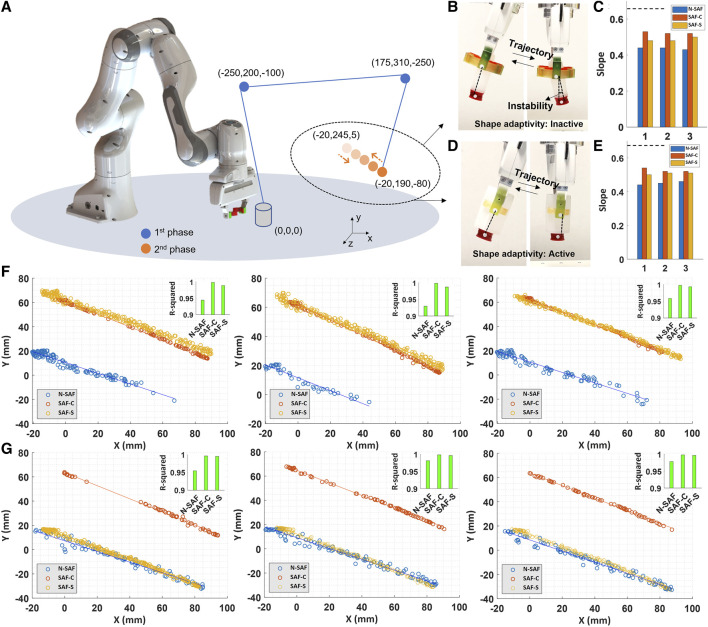
Grasp stability analysis using conventional fingertips (N-SAF) in blue, curved (SAF-C) and straight (SAF-S) shape-adaptive fingertips in red and yellow, respectively. **(A)** Trajectory of the manipulation in two different phases. The location of the coordinate points is just schematic and the exact trajectory can be seen by the numbers of the coordinate points. **(B)** An example of the second phase manipulation of the small object using the conventional fingertips. **(C)** The slope of the second phase manipulation of the small object using the three different fingertips. The tests were done for three times. The dashed line shows the slope of the trajectory of the robot **(D)** An example of the second phase manipulation of the large object using the curved shape-adaptive fingertips. **(E)** The slope of the second phase manipulation of the large object using the three different fingertips. The tests were done for three times. The dashed line shows the slope of the trajectory of the robot. **(F)** and **(G)** second phase data point manipulation of the small and large cylinders for three times, respectively. Using shape-adaptive fingertips, the objects are more stable during the defined repetitive linear trajectory of the robot, and the tip of the objects follows a quasi-linear path, as seen in red and yellow graphs, as well as having higher R-squared values. The videos are available in the Supplementary Material.

The proposed fingertip designs do not contribute to holding the object against gravity. This force is rather provided by the push of the parallel gripper. However, these designs and the improvements in enveloping the items not only distribute the loads and prevent local damage, but also stabilize them when manipulated fast. To prove this, we produced two soft hollow cylinders out of dragon skin 20, with wall thickness of 3 mm and diameters of 30 mm and 50 mm as two sample objects (He et al., 2020). To increase inertia, a weight of 25 gr was attached to the objects. The inner pressure of the cylinders was measured by a Honeywell SSCDANN015PGAA5 pressure sensor to keep the grasping force equal in all tests (a gauge pressure of 6 kPa was set for all the experiments). The shapes of the curved and the straight fingertips were adapted to the shape of the cylinders via the explained shape-adaptation process. Note that two hard cylinders with the same diameters as the objects were used for shape programming of the straight fingertips. The straight fingertips, which were not subjected to the shape programming step, were selected as conventional fingertips to standardize the friction coefficient for all tests. The Franka robot was programmed to first pick up the objects from the middle, move them between three coordinates, first phase in Figure 9A, and then perform a repeating linear movement for 10 times, second phase in Figure 9A. The trajectory slope of the second phase movement of the robot is 0.64. The manipulation (both phases) was performed with 75% of the maximum speed of the Franka robot. The stability analysis was focused more on the second phase of the manipulation, where the tip trajectory of the objects was recorded according to the method in section III.B, and compared with the trajectory of the robot. Figure 9B shows an example of the second phase manipulation of the small object. As seen, in case of using the conventional fingertips, the object rotates in the gripper and is not maintained securely. In Figure 9C, the slope of the tip trajectory of the small object is compared in three times of manipulations using the three different fingertips. The blue color is for conventional non-shape-adaptive fingertips, red for the curved shape-adaptive fingertips and yellow for the straight shape-adaptive fingertips. A linear regression model was fitted on the data points of the trajectory of the tip of the object and the slopes were acquired. As seen, the red and the yellow bars show closer numbers to the trajectory slope of the robot (0.64). This means that with the adaptive fingertips, the object is more secured in the grip and better follows the path of the robot. Figure 9C shows an example of the second phase manipulation of the large object using the adaptive fingertips. Similar to Figures 9C,E presents the results of the slope of the tip of the large object during three manipulations with the three different fingertips. It is again seen how the adaptive fingertips provide a more stable grasp compared to the conventional fingertips.

Another way to analyse the data is to study the linearity of the tip trajectory of the objects during the second phase manipulation. If the gripper securely maintains the objects, their tips exhibit a linear motion as well. Figures 9E,F depict the data points of the small and large objects in three times of manipulations with the three different fingertips, respectively. The trajectory of the objects manipulated by curved and straight SMP-SAFs is more linear than using the conventional fingertips. The data points are less scattered and the fitted linear model shows higher R-squared values. However, the manipulation using the conventional fingertips is less stable, the object vibrates more (Figure 9C), and the data points are more scattered. Another point that can be seen in the data is the relative drop of the objects during the first phase of the manipulation (Figure 9A). In Figure 9F the blue graphs which are attributed to the manipulation with the conventional fingertips are lower than the two other graphs. This shows that the small object was moved in line with the gravity during the first phase of the manipulation. In case of the large object (Figure 9G), this phenomenon was happened for both the manipulation with the conventional and the straight shape-adaptive fingertips. In conclusion, the curved shape-adaptive fingertips outperformed than the straight shape-adaptive fingertips in manipulation of the cylindrical objects. Also, both of the mentioned shape-adaptive fingertips showed a better performance than the conventional fingertips (Supplementary Video S1). It should be note that these results and analysis are just for the matter of comparison of the function of the fingertips. Certainly, with increasing the gripping load of the Franka robot hand, the grasp could be more stable.

## 4 Discussion

Diversity of objects poses a significant challenge for robotics manipulation. In this study, we utilized a vitrimeric thiol-acrylate resin to produce shape-adaptive fingertips for a two-finger industrial gripper. We introduced two different designs that offer solutions for effectively grasping objects of varying shapes, hardness, and fragility. The fingertips adapt to the outer surface profile of the objects via the conventional shape programming cycle of shape memory materials. This results in a better load distribution and a reduced risk of local damage to the objects. Furthermore, having more distributed contact points allows for the enhanced conformity which is particularly important during fast manipulation where failure of the grasp is a potential risk.

Thanks to the Joule-effect of the embedded electrically conductive wires and the parallel gripper, both the thermal stimulation and the force needed for each shape programming of the fingertips are performed without human intervention. However, this programming is still time-consuming. The reason for this originates from the poor thermal conductivity of polymers, which may take several minutes to cool the fingertips down for temporary shape fixing (heating the fingertips by the embedded wires takes 10–15 s while colling from above T_g_ to room temperature takes 4–5 min). As such, the application of the presented technology is still limited to cases where the shape of the objects does not change in each grasp, but rather from batch to batch. Furthermore, the fingertips can only adapt to the outer surface profile of the objects, and therefore cannot fully adapt to non-convex shapes. The shape adaption requires contact between the fingertips and the objects. Consequently, when dealing with temperature-sensitive objects, a sacrificial one must be used for shape-programming of the fingertips.

To widen the application of the presented fingertips and accelerate the shape transition process different steps can be taken into account. A first way would be to develop a composite shape memory polymer material by the addition of carbon-based fillers. This can increase the thermal conductivity of the material and results in a faster shape transition. In addition to the increase in thermal conductivity, the material would become electrically conductive which opens the possibility to exploit Joule effect for self-heating of the fingertips. A second point we will set out to tackle is the topology/geometrical optimization of the fingertips. Having a porous structure facilitates the flow of air through the fingertips and speeds up the cooling phase of the shape transition process. The topology optimization can also be employed with the goal of increasing the adaptivity, while retaining the shape fixity and shape recovery efficiencies. A third aspect to consider is the increase in the glass transition temperature (T_g_) which can accelerate the process of fixing the shape of a material. For instance, considering fingertips that are operated at room temperature, the time required for the material to cool from above the T_g_ to below the T_g_ is shorter if the T_g_ is increased. However, it is important to consider the impact of the increased T_g_ on the mechanical properties of the fingertips. Therefore, it is essential to balance the benefits of accelerating the shape-fixing process with the potential drawbacks of any changes in the mechanical properties.

It is worth to consider that fingertips may get damaged, as they are in direct contact with objects and under many programming cycles. As a result, being made of self-healing materials can extend the longevity of the fingertips.

## Data Availability

The original contributions presented in the study are included in the article/Supplementary Material, further inquiries can be directed to the corresponding author.
